# LINC00162 silencing enhances sorafenib sensitivity and inhibits thyroid cancer cells progression through modulation of MAPK signaling and apoptosis

**DOI:** 10.1038/s41598-025-12805-x

**Published:** 2025-08-13

**Authors:** Maryam Hejazi, Seyedeh Zahra Bahojb Mahdavi, Saba Abedimanesh, Ramin Heshmat, Bagher Larijani, Amir Ali Mokhtarzadeh, Gita Shafiee, Seyed Mohammad Tavangar

**Affiliations:** 1 Chronic Diseases Research Center, Endocrinology and Metabolism Population Sciences Institute, Tehran University of Medical Sciences, Tehran, Iran; 2 Immunology Research Center, Tabriz University of Medical Sciences, Tabriz, Iran; 3 Endocrinology and Metabolism Research Center, Endocrinology and Metabolism Clinical Sciences Institute, Tehran University of Medical Sciences, Tehran, Iran; 4 Department of Pathology, Dr. Shariati Hospital, Tehran University of Medical Sciences, Tehran, Iran

**Keywords:** LINC00162, Thyroid Cancer, Sorafenib, Chemosensitivity, MAPK pathway, Apoptosis, Autophagy, LncRNA, Cancer, Cell biology

## Abstract

Many studies have reported the aberrant expression of lncRNAs and indicated their role in cancer progression and drug resistance across various cancers. In this study, we aimed to evaluate the effect of LINC00162 lncRNA on the chemosensitivity of thyroid cancer cells, both individually and in combination with sorafenib, on various biological processes. In this regard, we conducted our experiments in several groups: (1) LINC00162 siRNA-transfected cells, (2) Sorafenib-treated cells, (3) Cells that received both siRNA transfection and sorafenib treatment (4) Control group. MTT assay results revealed that siRNA-mediated silencing of LINC00162 reduced the viability of the B-CPAP thyroid cancer cells and increased the sensitivity of these cells to sorafenib by reducing its IC50. Flow cytometry analysis of apoptosis and cell cycle progression indicated that LINC00162 silencing induced apoptosis and Sub-G1 cell cycle arrest, while its combination with sorafenib significantly increased the apoptosis rate and also arrested cells in the G2-M phase in addition to the Sub-G1 phase. This combination treatment increased the expression of apoptosis-related genes BAX, CASP3, CASP9 while decreasing BCL2 expression. Additionally, significant inhibition of the cell-cycle related genes MYC and Cyclin D and upregulation of TP53 were observed following combination treatment. Furthermore, the combination therapy reduced the migration of B-CPAP cells through the downregulation of MMP-3 and MMP-9. Colony sizes and numbers also decreased following siRNA-mediated silencing of LINC00162 and sorafenib treatment. qRT-PCR analysis of stemness-related genes, including NANOG, SOX2, CD44, and CD133 confirmed the findings of the colony formation assay. To understand the underlying mechanisms of LINC00162 lncRNA in thyroid cancer progression, we evaluated the expression of MAPK pathway genes. Our findings indicated that LINC00162 silencing, in combination with sorafenib, reduced the expression of MAPK, KRAS, and RAF genes. From our findings, we can conclude that LINC00162 silencing, both individually and combined with sorafenib, reduced the progression and viability of thyroid cancer cells through modulating genes involved in key pathways and could be considered a new therapeutic approach for the treatment of papillary thyroid cancer (PTC).

## Introduction

Thyroid cancer, ranked as the 9th most prevalent cancer, accounted for 586,000 cases globally in 2020^1^. The incidence rate of thyroid cancer in affluent and middle-income countries has steadily increased from 1998 to 2012^2^. Differentiated thyroid cancer (DTC), a subtype of thyroid cancer, consists of papillary thyroid cancer (PTC) and follicular thyroid cancer (FTC)^3^. Common tumor treatments, such as radiotherapy, surgery, and radioactive iodine (RAI), are used in DTC patients^4^. Among the various histological subtypes of thyroid cancer, PTC, which accounts for nearly 90% of cases, has the highest incidence compared to other subtypes. Despite the low mortality rates associated with PTC, its aggressive nature leads to recurrence and metastasis, potentially resulting in death^5–7^. Therefore, investigating the underlying mechanisms and developing novel therapies is essential for thyroid cancer treatment.

Increased activation of the mitogen-activated protein kinase (MAPK) pathway is observed in various cancers, including PTC. Point mutations in the RAS and BRAF genes, which activate the MAPK pathway, have been identified in two-thirds of PTC. The kinase cascades involving RAS–RAF–MEK and ERK are elevated following MAPK activation^8^. In this regard, novel therapies targeting the MAPK pathway could enhance the survival of patients. Sorafenib (Nexavar, BAY 43-9006) is a novel molecular-targeted therapy currently used for the treatment of thyroid cancer. The efficacy of sorafenib in thyroid cancer patients was demonstrated in two phase II clinical trials^9,10^. Sorafenib functions as a multikinase inhibitor, targeting serine/threonine kinases such as RAF, as well as targets VEGF receptors (VEGF-R) 1 to 3, platelet-derived growth factor receptor (PDGFR), RET, and c-KIT^11,12^. However, sorafenib only improves the progression-free survival rate by 5 months compared to the placebo in patients with radioactive iodine-refractory, locally advanced, or metastatic differentiated thyroid cancer. Also, adverse effects, particularly hand-foot skin reaction, led to a decrease in the drug dose, interruptions, and discontinuation of clinical trials. Other side effects of sorafenib include diarrhea, fatigue, alopecia, and weight loss^13^. In this context, combining sorafenib with other molecular agents and targeted therapies may enhance efficiency and reduce the required dose of chemotherapy drugs, potentially improving treatment outcomes.

Noncoding RNAs (ncRNAs), which constitute 90% of the transcriptome, cannot encode proteins. Based on their sizes, ncRNAs are categorized into two types: short ncRNAs (including small interfering RNA, PIWI-interacting RNAs, and miRNAs) and long ncRNAs (LncRNAs). ncRNAs are involved in various biological processes, including cell differentiation, inflammation, glucose metabolism, and different types of cancer^14–16^. LncRNAs, which are over 200 nucleotides in length, can regulate the activity and binding of transcription factors. They also modulate the stability of mRNA, either directly or by interacting with miRNAs and preventing their binding to mRNA^17^. Dysregulated expression of lncRNAs has been observed in various cancers. Their altered expression can act as an oncogene or contribute to tumorigenesis and the development of malignancies^18^. Various studies have investigated the role of different lncRNAs in thyroid cancer, reporting their involvement in oncogenesis, metastasis, apoptosis, and invasion^19,20^. For instance, a study demonstrated the prognostic role of the MIAT lncRNA in PTC, revealing that MIAT lncRNA enhances PTC cell invasion through the miR-150/EZH2 signaling pathway^19^. Also, overexpression of the IQCH-AS1 lncRNA, which is downregulated in doxorubicin-resistant thyroid cancer cells, sensitized these cells to doxorubicin^21^. In 2016, Piipponen et al. introduced lincRNA PICSAR (P38 Inhibited Cutaneous Squamous cell carcinoma-associated lincRNA), also known as LINC00162. They found that this previously uncharacterized lncRNA is overexpressed in keratinocyte-derived cutaneous squamous cell carcinoma. They reported that lncRNA PICSAR promotes cSCC progression through the ERK1/2 pathway, and knockdown of this lncRNA inhibited the migration and invasion of the cSCC cells^22^. Furthermore, it was reported that PICSAR enhances the expression of the PI3K/AKT/mTOR pathway by targeting miR-588, promoting hepatocellular carcinoma^23^. Moreover, the role of the lncRNA LINC00162 in various malignancies, including pancreatic cancer, hepatocellular carcinoma, and bladder cancer, has been demonstrated^23–25^. However, to the best of our knowledge, the role of the lncRNA LINC00162 has not yet been studied in thyroid cancer cells. In our previous study, bioinformatic analysis and tissue data revealed an increased expression of LINC00162 in thyroid cancer tissue. These findings are currently under review for publication and will be available in the near future. However, there is no study on the role of the lncRNA LINC00162 in thyroid cancer and its role in the chemosensitivity to chemotherapy drugs.

**Fig. 1:**
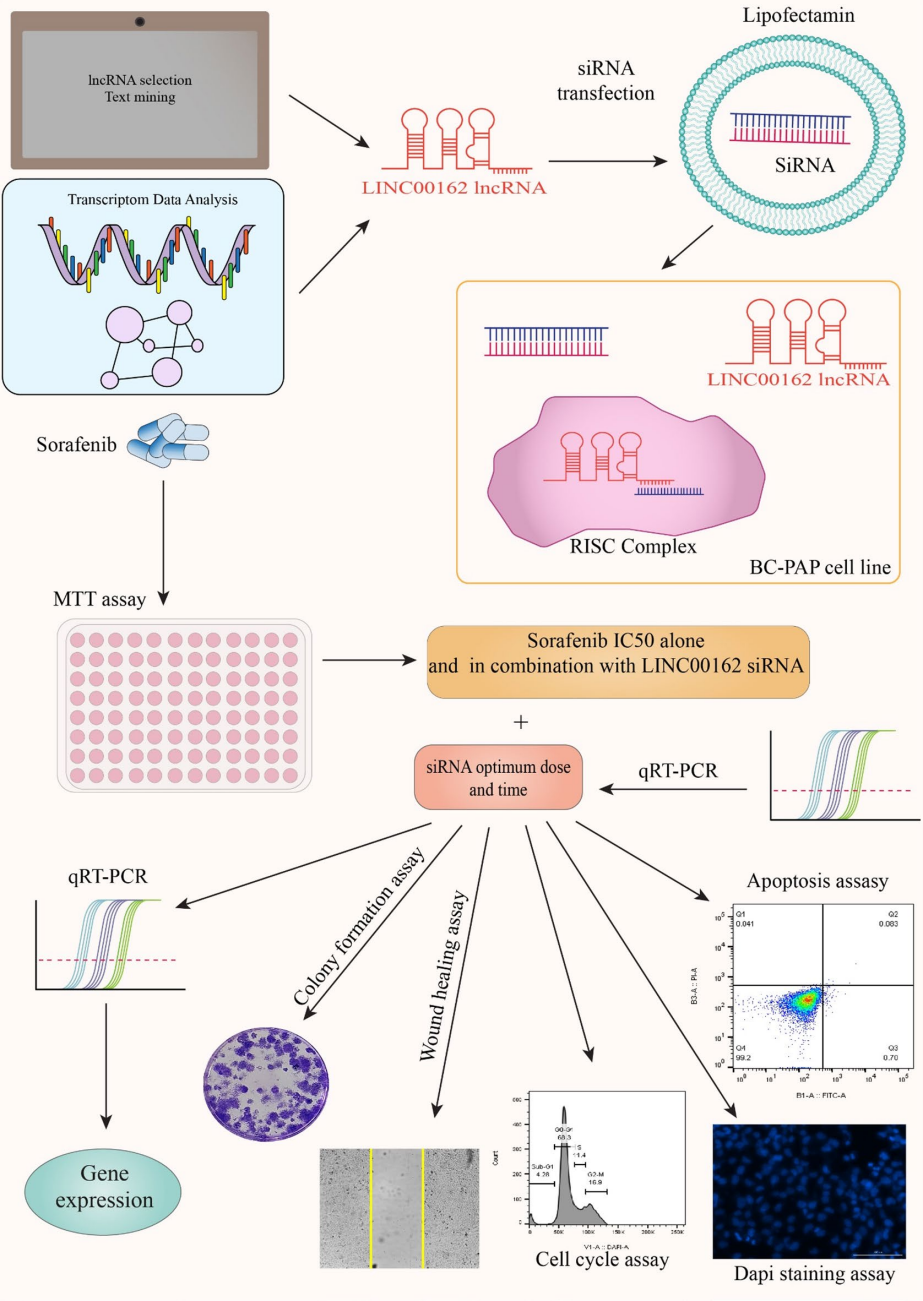
We selected the LINC00162 lncRNA, which is highly expressed in thyroid cancer, for our experiments. After transfecting LINC00162 siRNA using Lipofectamine, the optimum dose and transfection time were determined. Additionally, the IC50 of sorafenib alone and in combination with LINC00162 siRNA were assessed using the MTT assay. In this study, we examined the effects of LINC00162 silencing combined with sorafenib on cell cycle, apoptosis, colony formation ability, invasion, and the expression of the involved genes in the BCPAP cell line.

Thus, in the present study, we first investigated the effect of silencing lncRNA PICSAR on thyroid cancer cell viability. Next, we examined its role in the sensitivity of the thyroid cancer cells to sorafenib. Finally, we investigated the effect of LINC00162 silencing, sorafenib treatment, and combination therapy on the progression, apoptosis, cell cycle, and invasion of the B-CPAP thyroid cancer cells, while also evaluating the expression of genes involved in these pathways. Furthermore, we analyzed the expression of stemness-related genes and genes involved in the MAPK pathway. Figure 1 serves as the graphical abstract, providing a summary of the study. Results obtained from our study revealed that lncRNA LINC00162 silencing increases the sensitivity of B-CPAP cancer cells to sorafenib. Also, the combination of the LINC00162 siRNA and sorafenib reduces the viability, progression, and invasion of cancerous cells. Taken together, lncRNA PICSAR silencing can be used individually in treating thyroid cancer; additionally, by enhancing the chemosensitivity of thyroid cancer cells, combination therapy can be considered a new therapeutic intervention in thyroid cancer treatment.

## Materials and methods

### Cell culture

The B-CPAP cancer cell line of human papillary thyroid cancer was purchased from the National Cell Bank of Iran (Pasteur Institute, Tehran, Iran). The cell line was cultured in RPMI 1640 (Gibco, USA) enriched with 10% FBS (Gibco, USA) and penicillin/streptomycin (100 IU/ml and 100 µg/ml respectively). The cells were maintained in an incubator (37 °C, 5% CO2, 95% humidity). After reaching 70% confluency, the cells were subcultured using 0.25% trypsin-EDTA (Gibco, USA).

### Transfection efficiency

The cells were seeded in a 6-well plate at a density of 2.5 × 10^5^ cells per well. Following the manufacturer’s protocol, siRNA labeled with FITC was transfected into the seeded B-CPAP cells using Lipofectamine 3000 (Thermo Fisher Scientific). The efficiency of the transfection was evaluated by flow cytometry (Analyzer 10, Miltenyi Biotec, Germany).

### Dose and time optimization of the SiRNA

The cells were seeded in a 6-well plate at a density of 2.5 × 10^5^ cells per well. After 24 hours, the various doses of 10, 20, 40, and 60 pmol of LINC00162 siRNA targeting the lncRNA, synthesized by BIONEER company (Table 1), and negative control siRNA (Negative control, 5’ UUCUCCGAACGUGUCACGUUU 3’), siRNA guide strand as a scramble with no target) transfected into cultured cells using Lipofectamine 3000 (Thermo Fisher Scientific) and incubated for 6 h, following the manufacturer’s protocol. Complete media containing 10% FBS was added to each well six hours later. To determine the optimum dose and time of the transfection, the levels of LINC00162 were evaluated after 24, 48, and 72 h of transfection by quantitative reverse transcription polymerase chain reaction (qRT-PCR).

**Table 1:** SiRNA LINC00162 sequence.

LIINC00162	Sense	CUCAGACAUCUGCAGUCACUUCACA
	Antisense	UGUGAAGUGACUGCAGAUGUCUGAGGA

### RNA extraction and qRT-PCR

Total RNA from the B-CPAP cells was isolated by the Trizol RNA extraction kit (GeneAll, Korea). The purity and concentration of the isolated RNAs were determined with a NanoDrop spectrophotometer (Thermo Fisher Scientific Life Sciences, USA) by measuring absorbance at 260 and 280 nm. A reverse transcription kit (AddScript cDNA Synthesis Kit) was used to synthesize Complementary DNA (cDNA) from 1 µg of the isolated RNA. The isolated RNA was reverse transcribed into cDNA using a Thermal cycler (Bio-Rad, USA).

qRT-PCR was conducted using a qRT-PCR system (Roche, Switzerland) and the relative mRNA expression of the lncRNA LINC00162, BAX, BCL2, CASP3, CASP9, TP53, MYC, MAPK, RAS, RAF, NANOG, SOX2, CD44, CD133, MAP1LC3B, ATG5, and ATG7 was evaluated (Ampliqon™ RealQ plus 2X Real-Time PCR Master Mix, Denmark) and calculated using the 2^−ΔΔCt^ method. The expression of the LINC00162 lncRNA and genes was normalized to GAPDH. The sequence of primers used in this experiment was blasted using the NCBI’s Primer-BLAST (Table 2).

**Table 2:** Primer Sequences.

Primers	Sequences
BAX	Forward: 5′ GACTCCCCCCGAGAGGTCTT 3′ Reverse: 5′ ACAGGGCCTTGAGCACCAGTT 3′
BCL2	Forward: 5′ CTGTGGTCCACCTGACCCTCCGC 3′ Reverse: 5′ CGTACAGTTCCACAAAGGCATCCCAGC 3
CASP3	Forward: 5′ GGAAGCGAATCAATGGACTCTGG 3′ Reverse: 5′ GCATCGACATCTGTACCAGACC 3
CASP9	Forward: 5′ CCAGAGATTCGCAAACCAGAGG 3′ Reverse: 5′ GAGCACCGACATCACCAAATCC 3′
TP53	Forward: 5′ CCTCAGCATCTTATCCGAGTGG 3′ Reverse: 5′ TGGATGGTGGTACAGTCAGAGC 3′
Cyclin D	Forward: 5′ TGCTCTAGATGAATTCTTATCCCCTGCCC 3′ Reverse: 5′ CGCGGATCCAAGAGAAGAGGGACACAGCC 3′
MYC	Forward: 5′ GCTGCTTAGACGCTGGATTT 3′ Reverse: 5′ CACCGAGTCGTAGTCGAGGT 3′
NANOG	Forward: 5′ CTAAGAGGTGGCAGAAAAACA 3′ Reverse: 5′ CTGGTGGTAGGAAGAGTAAAGG 3′
SOX2	Forward: 5′ ACATGTGAGGGCCGGACAGC 3′ Reverse: 5′ TTGCGTGAGTGTGGATGGGATTGG 3′
CD44	Forward: 5′ CAAGCCACTCCAGGACAAGG 3′ Reverse: 5′ ATCCAAGTGAGGGACTACAACAG 3′
CD133	Forward: 5′ GACCGACTGAGACCCAACATC 3′ Reverse: 5′ GGCTAGTTTTCACGCTGGTCA 3′
MAPK1	Forward: 5′ CCCAAATGCTGACTCCAAAGC 3′ Reverse: 5′ GCTCGTCACTCGGGTCGTAAT 3′
RAS (KRAS)	Forward: 5′ CTCCCTGTGTCAGACTGCTCTTT 3′ Reverse: 5′ GGCCTTGCAACCTTGGTCTCTTC 3′
RAF 1	Forward: 5′ TTTCCTGGATCATGTTCCCCT 3′ Reverse: 5′ ACTTTGGTGCTACAGTGCTCA 3′
MAP1LC3B	Forward: 5′ CGGAGAAGACCTTCAAGCAG 3′ Reverse: 5′ CTGGGAGGCATAGACCATGT 3′
ATG5	Forward: 5′ AAAGATGTGCTTCGAGATGTGT 3′ Reverse: 5′ CACTTTGTCAGTTACCAACGTCA3′
ATG7	Forward: 5′ TGCTATCCTGCCCTCTGTCTT 3′ Reverse: 5′ TGCCTCCTTTCTGGTTCTTTT 3′
LINC00162	Forward: 5′ GCTCTAACTCAGGGCTCCA 3′ Reverse: 5′ TGCTCCCCACCTAAGCAATG 3′
GAPDH	Forward: 5′ CAAGATCATCAGCAATGCCT 3′ Reverse: 5′ GCCATCACGCCACAGTTTCC 3′

### MTT assay

The half maximal inhibitory concentration (IC50) of sorafenib was determined by MTT assay. In this regard, B-CPAP cells were seeded at a density of 7 × 10^3^ cells per well. 24 h following the cultivation, seeded cells were treated with various concentrations of sorafenib ranging from 0.1 to 100 µg/ml. The next day, the media in each well were aspirated and 50 µL of MTT solution (5 mg/mL, Sigma-Aldrich, Germany) and 100 µL of complete media was added to each well, and the plate was incubated for 4 h. In order to dissolve formazan crystals, dimethyl sulfoxide was added to each well, and the plate was incubated for 30 min. The absorbance (OD) at 570–620 nm was measured by an ELISA microplate reader (Tecan, Switzerland). MTT assay was also performed to investigate the effect of LINC00162 silencing on the B-CPAP cells’ sensitivity to sorafenib. Thus, first, cells were transfected with LINC00162 siRNA and then treated with sorafenib. IC50 of the individual groups treated with sorafenib and the combination group were determined. Cell viability following LINC00162 siRNA transfection was also assessed with the MTT assay.

### Annexin/PI apoptosis assays

To investigate the effect of the LINC00162 siRNA, both individually and in combination with Sorafenib, on apoptosis induction in B-CPAP cell lines, an apoptosis assay was performed. First, B-CPAP cells were seeded at a density of 1.5 × 10^5^ cells per well and incubated for 24 h. LINC00162 siRNA was transfected into the cells, and 24 h later, the relevant groups were treated with sorafenib and incubated for an additional 24 h. Subsequently, the cells were harvested from each well and washed with PBS. The cells in each group were resuspended in binding buffer and stained using a V-FITC/PI staining kit (Immunostep, Spain), according to provided protocols. Apoptosis induction in each group was assessed using flow cytometry (Analyzer 10, Miltenyi Biotec, Germany). All experiments were conducted in triplicate.

### DAPI staining

Apoptosis induction based on chromatin fragmentation was investigated with 4′,6-diamidino‐2 phenylindole (DAPI) staining. Firstly, B-CPAP cells were seeded in a 96-well plate with a density of 7 × 10^3^ cells per well. The relevant groups were transfected with LINC00162 siRNA and subsequently treated with sorafenib. Then, the cells were fixed with 100 µL of 4% paraformaldehyde and incubated for 1.5 h. Fixed cells were washed with PBS multiple times, permeabilized with 0.1% Triton-X-100, and incubated for 15 min. After a wash step with PBS, the cells of each group were stained with 100 µl of DAPI (0.1%, Sigma-Aldrich, USA) and incubated for an additional 10 min in the dark. Morphological changes were observed using the DAPI channel of Cytation 5 fluorescence imaging system (BioTK).

### Cell cycle assay

The impact of the LINC00162 siRNA, both individually and combined with sorafenib, on the cell cycle progression of B-CPAP cells was examined by flow cytometry. First, cells were seeded in a 6-well plate at a density of 1.5 × 10^5^ cells per well. LINC00162 siRNA was transfected into cells, and after 24 h, the transfected groups were treated with sorafenib, and the plate was incubated for an additional 24 h. After the incubation period, the cells were harvested and fixed with cold 80% ethanol at -20 °C overnight. Then, fixed cells were centrifuged, resuspended in 500 µl of cold PBS containing 5 µL of RNase A, and incubated for 30 min. Incubated cells were centrifuged and resuspended in DAPI and Triton-containing PBS. After a 10-minute incubation, stained cells were centrifuged and washed with PBS. Cell cycle arrest in each group was analyzed using flow cytometry (Analyzer 10, Miltenyi Biotec, Germany).

### Colony formation assay

The inhibitory effect of siRNA-mediated LINC00162 lncRNA inhibition and treatment with sorafenib on the colony formation ability of B-CPAP thyroid cancer cells was investigated with the clonogenic assay. First, 2.5 × 10^3^ cells were seeded in a 6-well plate, transfected with LINC00162 siRNA, treated with sorafenib, and the plate was incubated for 10 days. Afterward, each group was washed with PBS, and paraformaldehyde (5%) was used to fix each group. The cells were then stained with crystal violet for 20 min, and the colonies were photographed.

### Wound healing assay

Cellular migration of the B-CPAP thyroid cancer cells following LINC00162 inhibition and treatment with sorafenib was assessed with the wound healing assay (scratch). B-CPAP cells were seeded in a 24-well plate with a density of 7.5 × 10^4^ cells per well for this purpose. LINC00162 siRNA was transfected into the seeded cells, and 24 h later, the relevant groups were treated with Sorafenib. A yellow pipette tip was employed to scratch the cellular monolayers, and the migration distance of the B-CPAP cells to the wound area was captured at 0, 24, and 48 h. The rate of the open wound area was calculated with Image J software.

### Statistical analysis

All data are expressed as mean ± standard deviation and *P* < 0.05 was considered statistically significant. Statistical analysis was conducted using GraphPad Prism 8.0. Flow cytometry data were analyzed by Flowjo, and wound healing assay images were analyzed with image J (version 1.54d). The differences between two groups and multiple groups were analyzed using Student’s t-tests and one-way analysis of variance (ANOVA), respectively.

## Results

### Efficient transfection of SiRNA

**Fig. 2:**
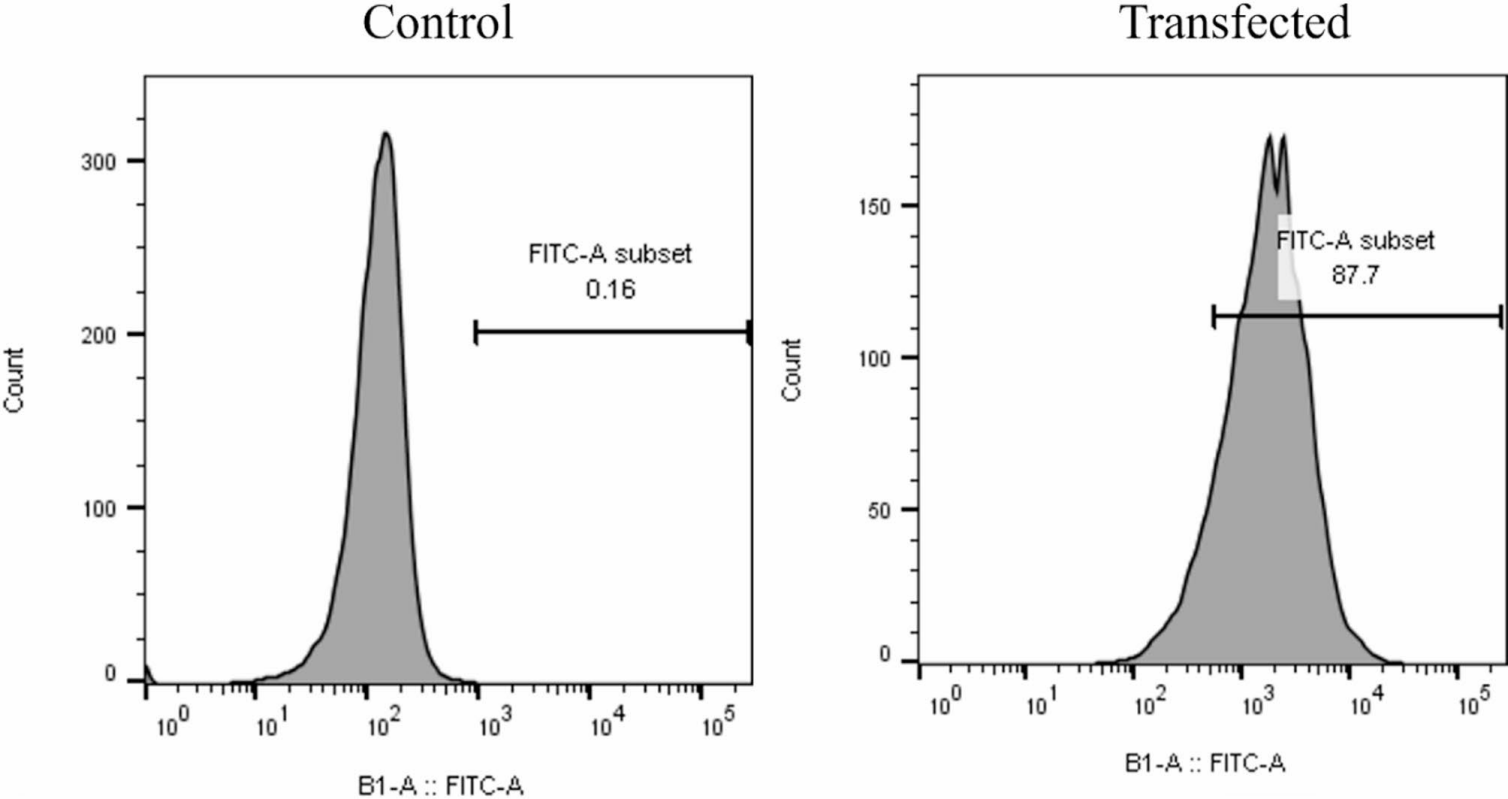
LINC00162 siRNA transfection. The FITC-labeled siRNA was successfully transfected into B-CPAP cells with an 87.7% transfection rate.

Flow cytometry data showed that FITC-labeled siRNA was successfully transfected into B-CPAP cells with 87.7% efficiency compared to the untransfected control group, demonstrating the effectiveness of Lipofectamine in delivering siRNA to B-CPAP cancer cells (Fig. 2).

### PICSAR SiRNA reduced expression of the LINC00162 LncRNA

**Fig. 3:**
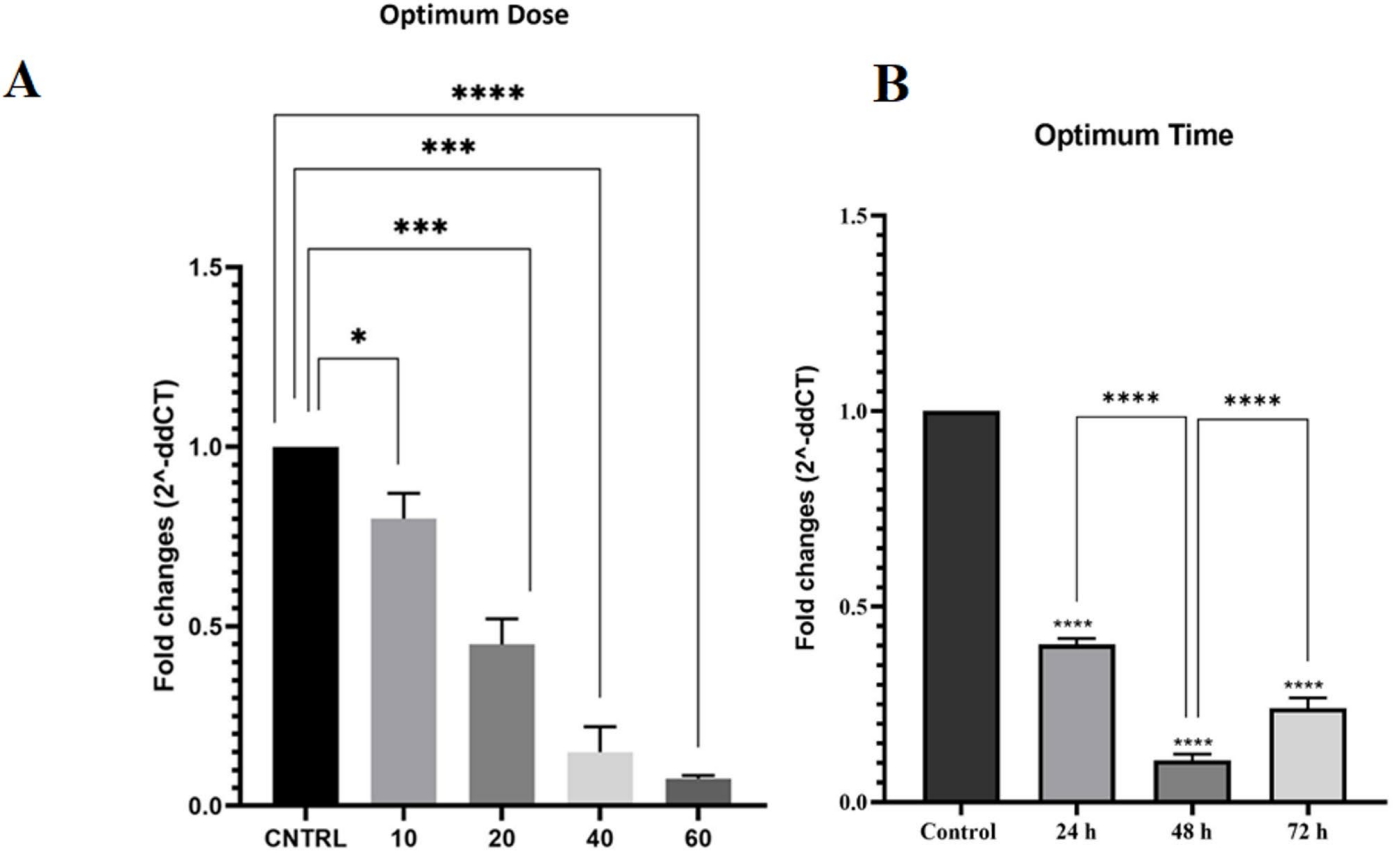
Determining (**A**) optimum dose and (**B**) time of the transfection.

In order to determine the optimal dose of the LINC00162 siRNA, B-CPAP cells were transfected with various doses of the siRNA (10, 20, 40, 60 pmol) for 24, 48, and 72 h. qRT-PCR results showed that the transfection of 60 pmol of the siRNA significantly reduced LINC00162 expression (Fig. 3A). Additionally, 48 h post-transfection, the expression of LINC00162 was markedly decreased compared to the control groups (Fig. 3B). Thus, 60 pmol was selected as the optimum dose, while 48 h was chosen as the optimum time of the transfection for the subsequent experiments.

### Silencing of the LINC00162 LncRNA reduced the viability of the cells

**Fig. 4:**
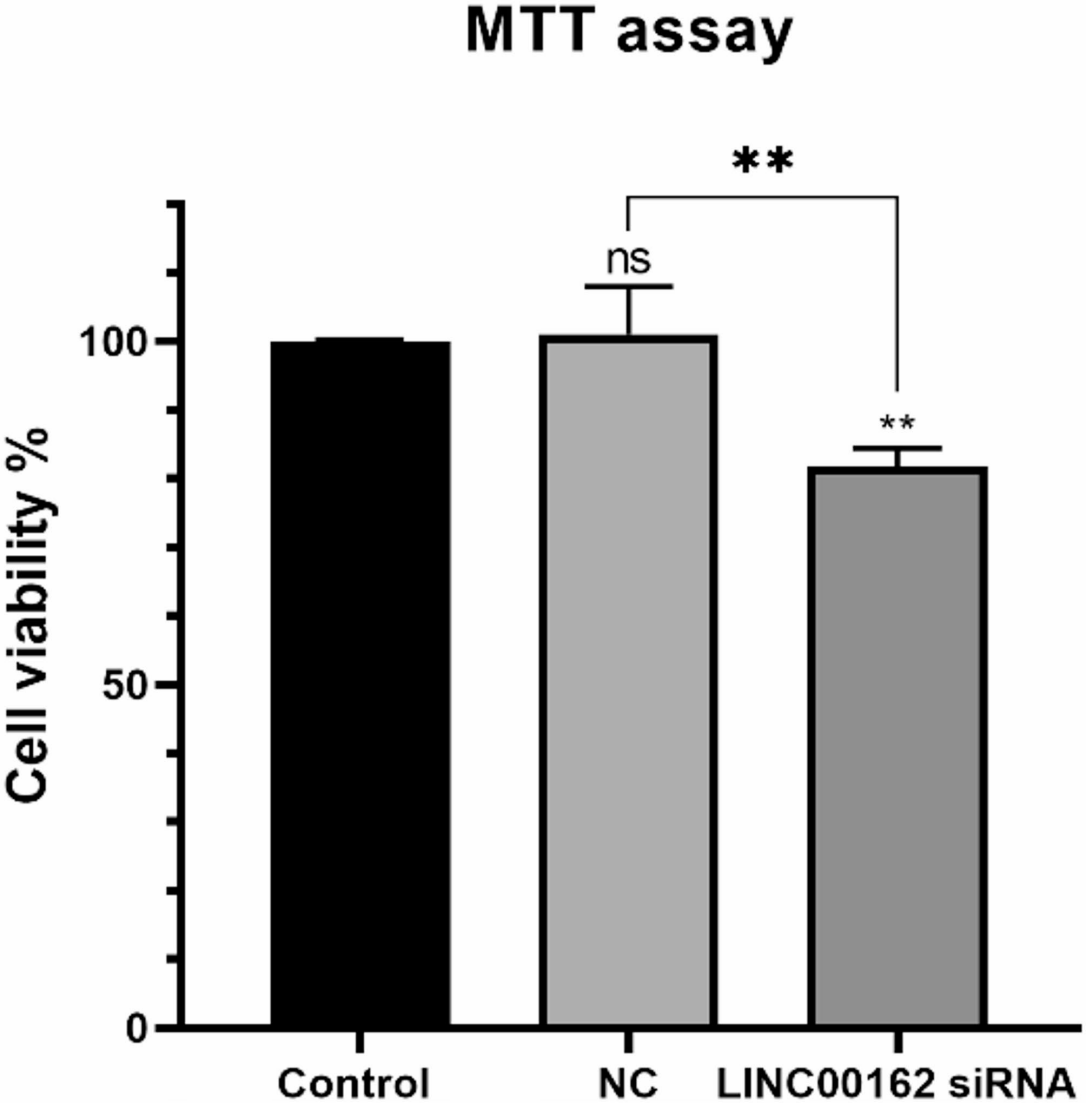
siRNA transfection significantly reduced the viability of B-CPAP cells compared to control and NC groups (**p < 0.01 and ns = not significant).

The MTT assay was used to determine the viability of the B-CPAP cells following siRNA-mediated silencing of the LINC00162 lncRNA. B-CPAP cells were transfected with the optimal dose of 60 pmol for 48 h The results showed no significant differences in the viability of the cells transfected with scramble siRNA compared to the untransfected control cells. Additionally, the MTT assay results showed a significant decrease in the viability of the cells transfected with LINC00162 siRNA compared to the control and NC groups (Fig. 4).

### Inhibition of LINC00162 LncRNAs increased sensitivity of B-CPAP cells to sorafenib

**Fig. 5:**
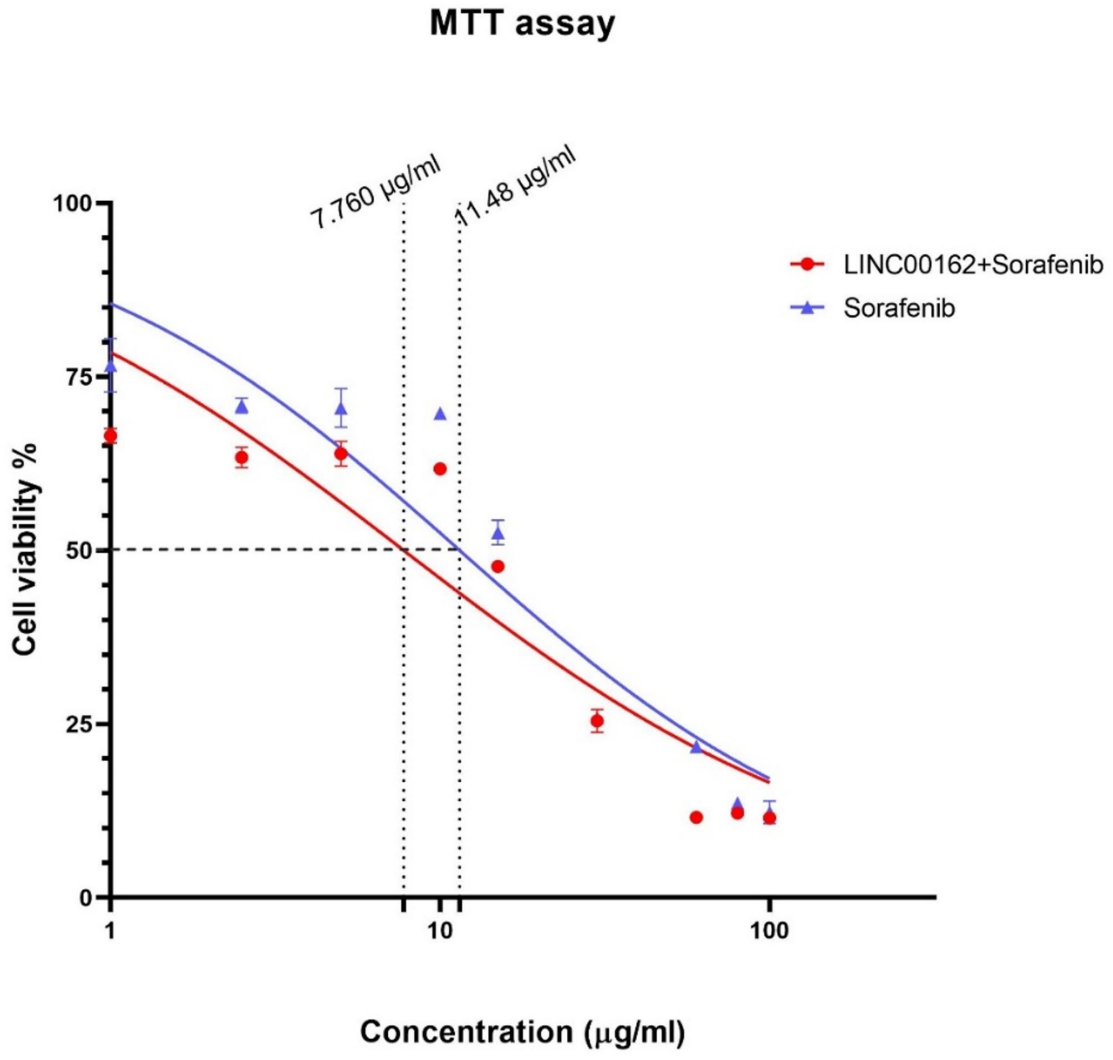
IC50 of the sorafenib individually and combined with LINC00162 siRNA transfection. siRNA-mediated silencing of the LINC00162 sensitized B-CPAP cells to sorafenib and reduced the IC50 value from 11.48 µg/ml to 7.760 µg/ml.

MTT assay was performed to determine IC50 of the sorafenib both individually and following LINC00162 inhibition. B-CPAP cells were treated with various concentrations of sorafenib ranging from 0.1 µg/ml to 100 µg/ml. The results showed that 11.48 µg/ml of sorafenib reduced the viability of the cells by 50% compared to the untreated control group. However, the IC50 of the sorafenib following LINC00162 siRNA transfection was determined to be 7.760 µg/ml (Fig. 5). This reduced IC50 of the sorafenib in the combination group indicates that LINC00162 silencing decreased the efficient dose of sorafenib by increasing the sensitivity of B-CPAP cells to the chemotherapy drug.

### siRNA-mediated inhibition of LINC00162 LncRNAs combined with sorafenib induced apoptosis

**Fig. 6:**
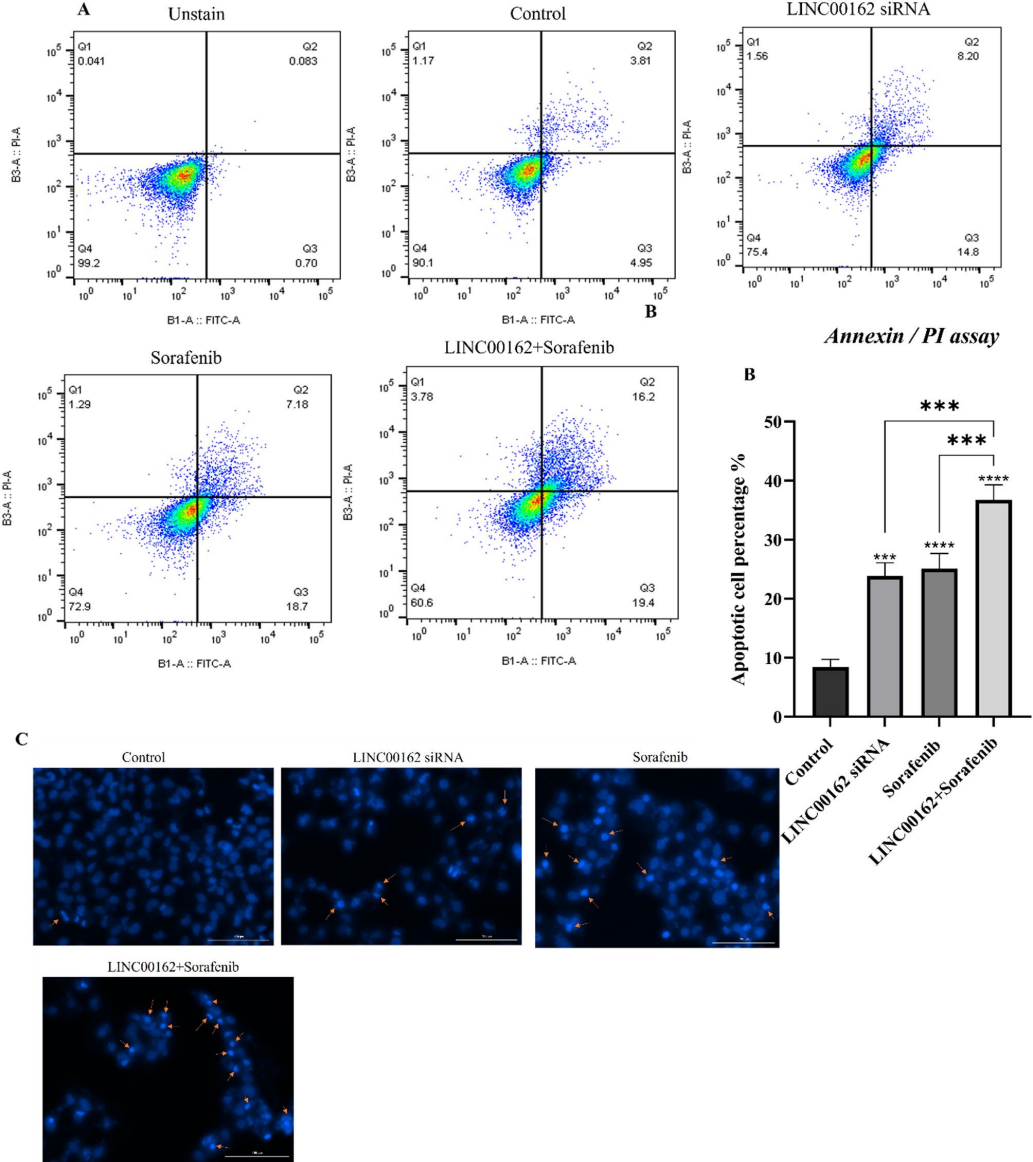
Effect of the LINC00162 silencing combined with sorafenib on apoptosis induction in B-CPAP thyroid cancer cells. (**A**) Apoptosis rate in various groups evaluated by AnnexinV/PI assay. (**B**) While LINC00162 siRNA transfection and treatment with sorafenib significantly increased apoptosis rate, the combination of these treatments induced apoptosis more than individual treatments (****p < 0.0001 and ***p < 0.001). (**C**) DAPI staining to determine chromatin fragmentation.

AnnexinV/PI assays were performed to assess the effect of LINC00162 inhibition and its combination with sorafenib on the apoptosis of B-CPAP cells. Flow cytometry results showed that apoptosis rate induced by LIN00162 siRNA and sorafenib treatment individually were 23% and 25.88%, respectively. This rate increased to 35.6% in the combination treatment group (Fig. 6A). These findings suggested that while individual treatments with LINC00162 inhibition and treatment with sorafenib significantly induced apoptosis compared to the control group (***p < 0.001 and ****p < 0.0001, respectively), apoptosis rate in the combination group with LINC00162 inhibition and sorafenib treatment was significantly higher than individual treatments and control group (***p < 0.001 and ****p < 0.0001, respectively) (Fig. 6B). A chromatin fragmentation assay with DAPI staining also confirmed apoptosis assay results and showed a higher number of apoptotic cells in the combination therapy group compared to individual treatments (Fig. 6C).

**Fig. 7:**
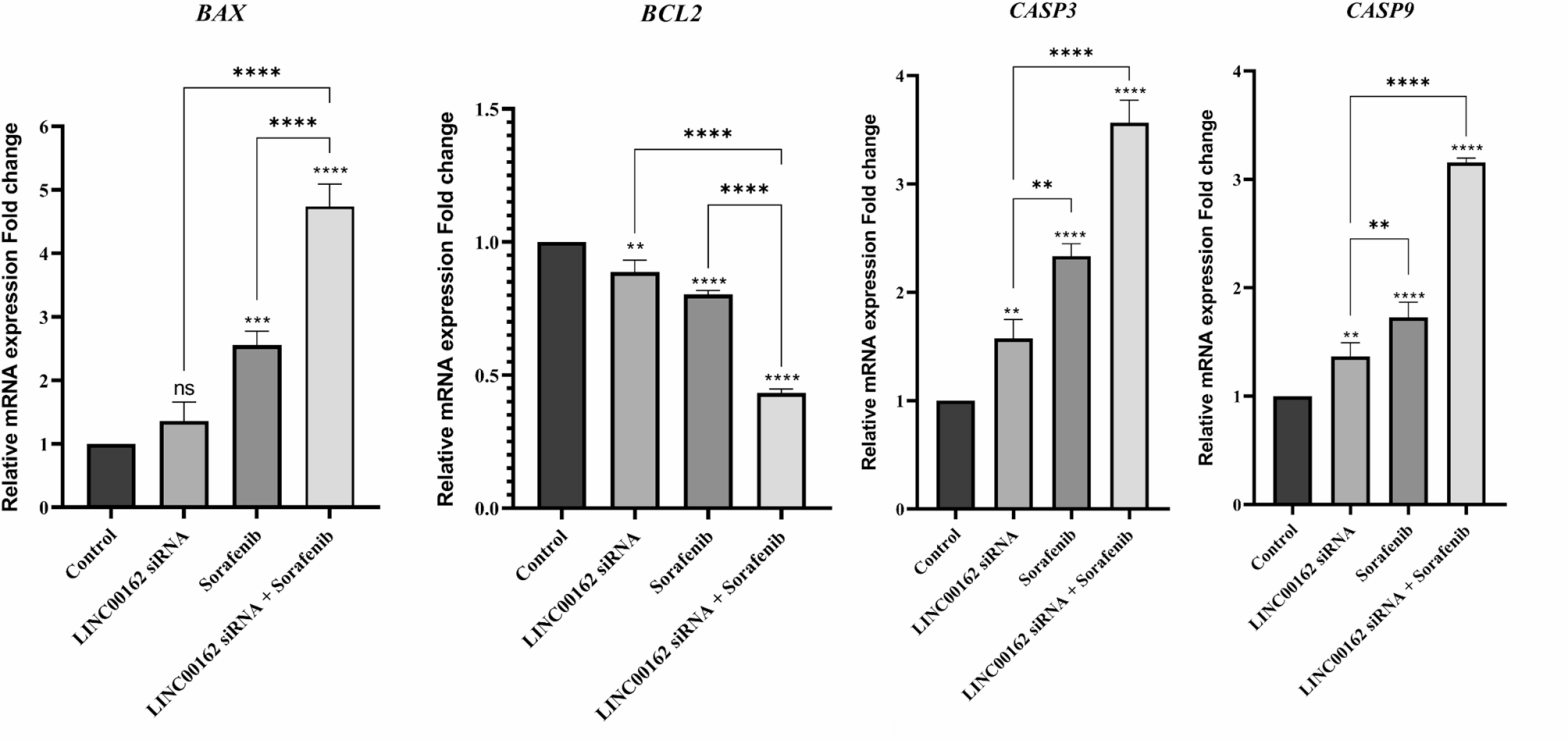
Comparing the combination group to the control group, qRT-PCR showed significant upregulations of Bax, caspase 3, and caspase 9 and downregulation of Bcl-2 (****p < 0.0001, ***p < 0.001, **p < 0.01, *p < 0.05; ns = not significant).

To confirm flow cytometry and DAPI staining assay results, relative mRNA expression of the genes involved in the apoptosis pathway, including BAX, BCL2, CASP3, and CASP9, were evaluated by qRT-PCR. The results indicated that while siRNA-mediated silencing of the LINC00162 lncRNA had no significant effect on the expression of the pro-apoptotic BAX gene, sorafenib treatment significantly increased the expression of the BAX (***p < 0.001) compared to the control group. Also, LINC00162 siRNA transfection followed by sorafenib treatment increased relative BAX mRNA expression more than individual treatments (****p < 0.0001). Furthermore, the qRT-PCR results indicated that LINC00162 silencing and sole treatment with sorafenib significantly elevated mRNA levels of the proapoptotic CASP3 and CASP9 genes while suppressing the expression of the anti-apoptotic gene BCL2. Furthermore, siRNA transfection combined with sorafenib treatment significantly upregulated pro-apoptotic CASP3 (****p < 0.0001) and CASP9 (****p < 0.0001) genes expression while downregulating the expression of the anti-apoptotic BCL2 compared to the individual treatments (Fig. 7).

### siRNA-mediated Inhibition of LINC00162 LncRNAs combined with sorafenib induced cell cycle arrest

**Fig. 8:**
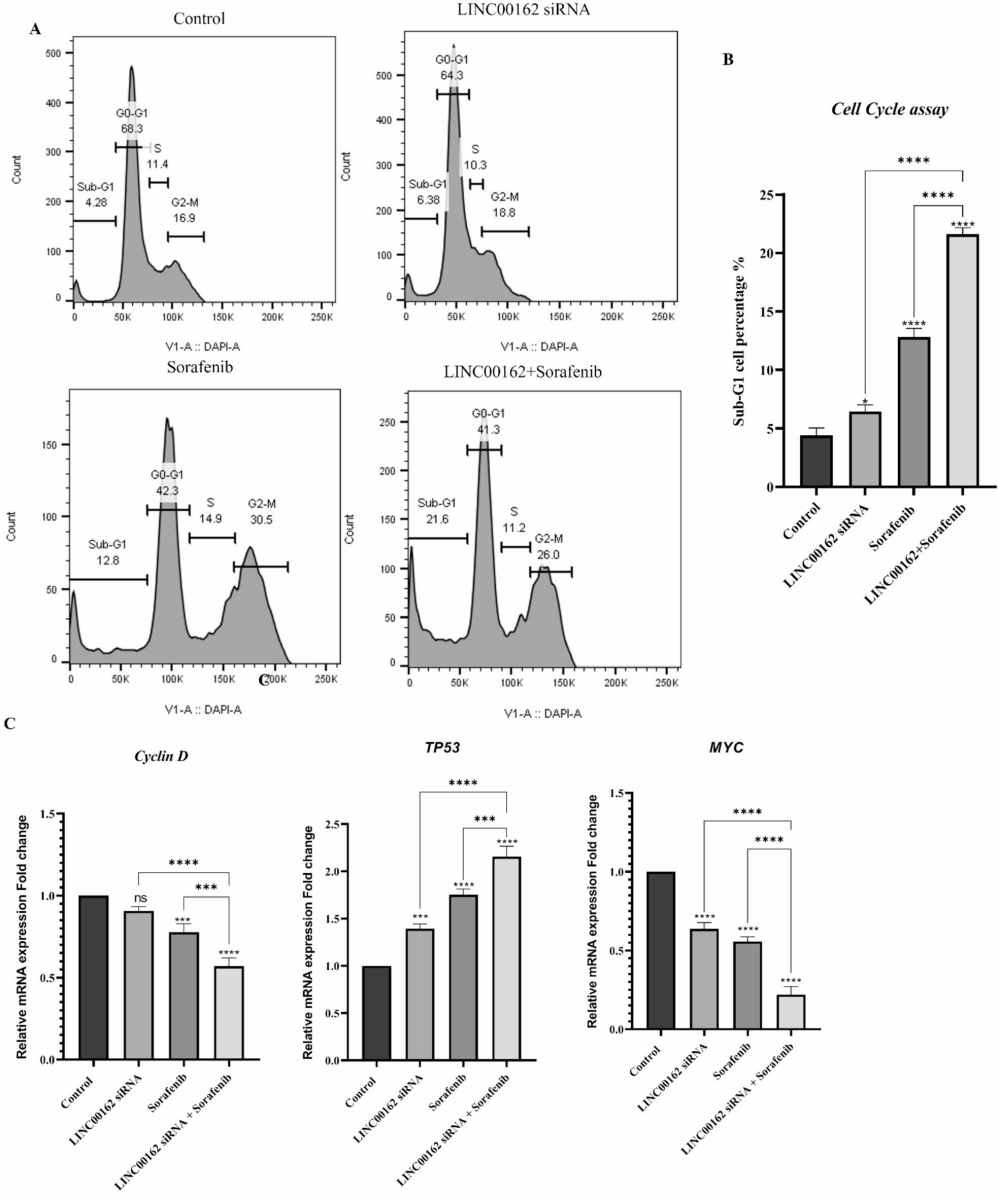
Analysis of the impact of siRNA-mediated silencing of LINC00162 and sorafenib on cell cycle progression in B-CPAP cells. **(A)** LINC00162 siRNA transfection increased Sub-G1 arrested cells while sorafenib increased Sub-G1 and G2-M arrested cells rate. The combination of these treatments increased both Sub-G1 and G2-M arrested cell rates. **(B)** The graph illustrates the distribution of cells across different phases within the treatment groups. **(C)** Analysis of TP53, Cyclin D, and MYC expression by qRT-PCR (****p < 0.0001, ***p < 0.001, and ns = not significant).

In this study, flow cytometry analysis was performed to determine how LINC00162 silencing and the combination of sorafenib affected B-CPAP thyroid cancer cell cycle progression and to evaluate cell cycle distribution. In the siRNA-transfected group, LINC00162 silencing in B-CPAP cells increased the percentage of cells in the Sub-G1 phase from 4.28% in the control group to 6.38%. Sorafenib treatment increased the rate of cells arrested in the Sub-G1 (12.8%) and G2/M (30.5%) phases. The LINC00162 silencing and sorafenib treatment combination increased the Sub-G1 arrested cell rate to 21.6%. Also, combination treatment increased cell distribution in the G2-M phase (26%) compared to the control group (16.9%). However, the rate of G2-M arrested cells that had been increased by sorafenib (30.5%) was slightly reduced in the combination group (26%) (Fig. 8A,B).

To further confirm the cell cycle assay findings, we evaluated the expression of the genes involved in the cell cycle progression. The qRT-PCR results showed that the expression levels of TP53 significantly increased (***p < 0.001) following LINC00162 silencing. In addition, sorafenib treatment significantly upregulated TP53 expression (****p < 0.0001) compared to the control group. With the combination of LINC00162 inhibition and sorafenib treatment, the relative mRNA expression of the TP53 increased significantly compared to the control group (****p < 0.0001) and individual treatments (Fig. 8C). These findings confirm the results of cell cycle assay.

To further investigate the role of LINC00162 lncRNA in cell cycle progression, we evaluated the expression of the MYC and cyclin-D genes, which are crucial in regulating cell cycle progression. qRT-PCR results showed that while LINC00162 inhibition (****p < 0.0001) and treatment with sorafenib (****p < 0.0001) reduced MYC expression in B-CPAP thyroid cancer cells, combination of these therapeutic methods significantly reduced of MYC expression compared to control (****p < 0.0001) and individual groups (****p < 0.0001). (Fig. 8C). Additionally, LINC00162 siRNA had no significant impact on the expression of Cyclin D. However, sorafenib treatment (***p < 0.001) and the combination of LINC00162 and sorafenib (****p < 0.0001) significantly reduced mRNA expression of Cyclin D.

### siRNA-mediated inhibition of LINC00162 lncRNAs combined with sorafenib inhibited the stemness ability of the B-CPAP cells.

**Fig. 9:**
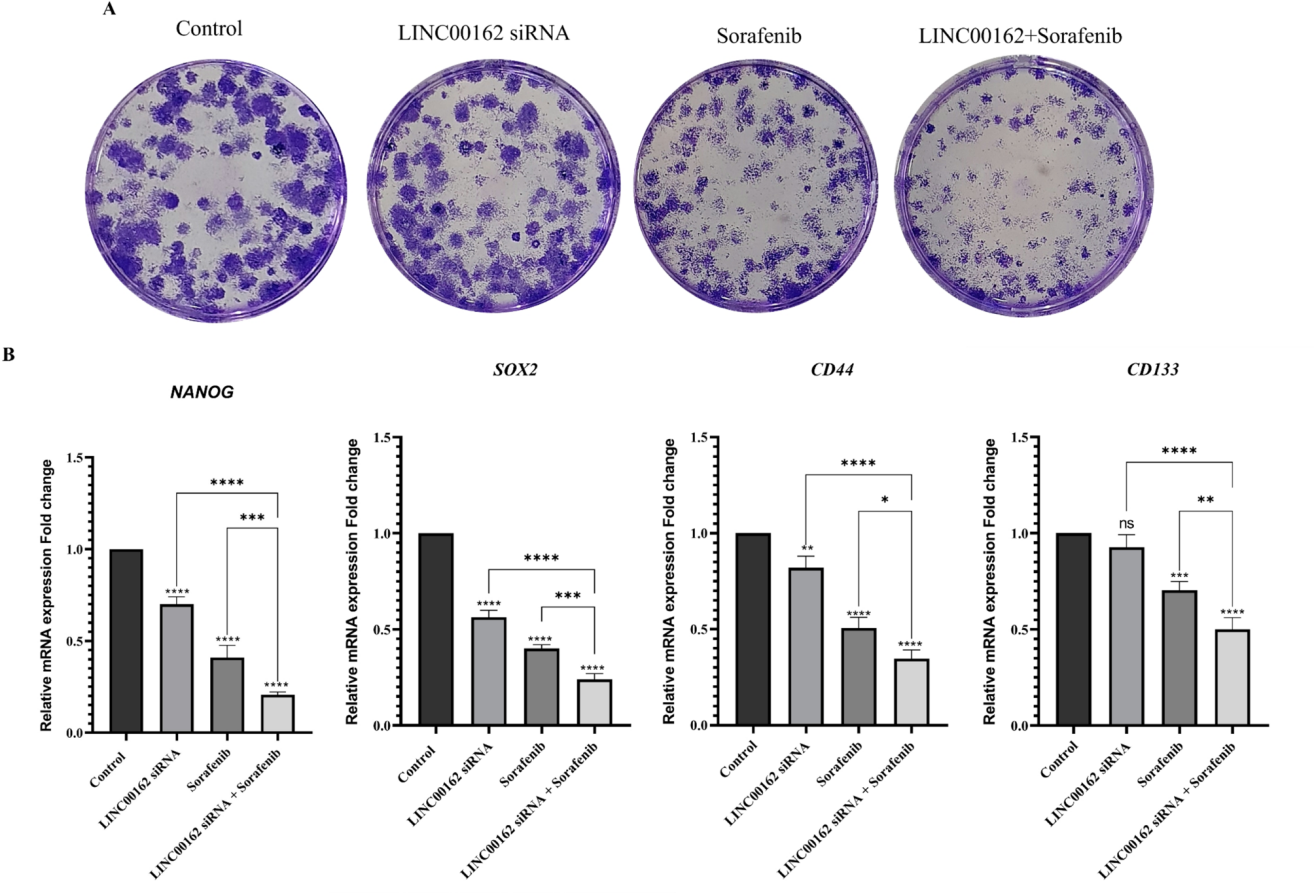
(**A**) Analysis of LINC00162 siRNA and sorafenib combination effect on colony formation of B-CPAP cells. (**B**) Analysis of NANOG, SOX2, CD44, and CD133 expression by qRT-PCR (****p < 0.0001,  ***p < 0.001, and ns = not significant).

Colony formation and stemness of B-CPAP cells were examined by colony formation assay. The results showed that while transfection with the LINC00162 siRNA slightly reduced the number of the formed colonies, sorafenib treatment decreased both the number and size of the colonies. Furthermore, the size and number of colonies in the group transfected with siRNA and treated with sorafenib significantly decreased compared to the control and individual treatment groups (Fig. 9A).

To evaluate the impact of combination treatment on the stemness of the B-CPAP cells, mRNA fold changes of the NANOG, SOX2, CD44, and CD133 genes, key regulators of the stemness and self-renewal of cancer cells, were investigated using qRT-PCR. The results indicated that in cells where LINC00162 was silenced through siRNA transfection, the expression of Nanog (****p < 0.0001), Sox2 (****p < 0.0001), and CD44 (**p < 0.01) significantly decreased compared to the control group. However, no significant downregulation was observed in the expression of CD133 following LINC00162 silencing. Sorafenib treatment also significantly downregulated expression of the NANOG (****p < 0.0001), SOX2 (****p < 0.0001), CD44 (****p < 0.0001, and CD133 (***p < 0.001). Furthermore, the qRT-PCR results demonstrated that combining the LINC00162 siRNA and sorafenib treatment led to a more pronounced reduction (****p < 0.0001) in the expression of the NANOG, SOX2, CD44, and CD133 genes than either treatment alone. These findings confirm the role of LINC00162 lncRNA in the stemness of the B-CPAP cancer cells (Fig. 9B).

### siRNA-mediated Inhibition of LINC00162 LncRNAs combined with Sorafenib inhibited the migration of the B-CPAP cells

**Fig. 10:**
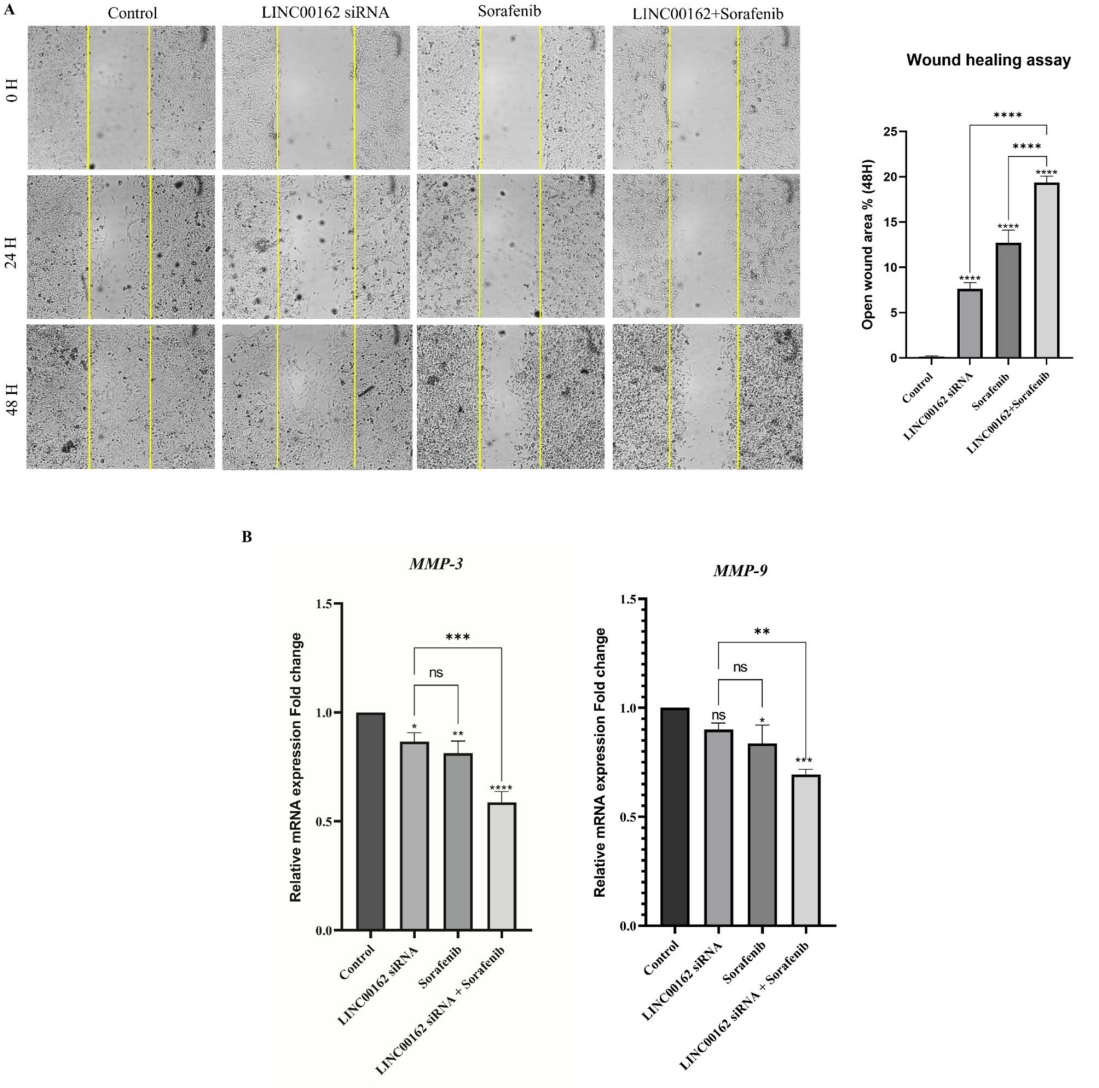
(**A**) Analysis of LINC00162 siRNA and sorafenib combination effect on migration of B-CPAP cells. (**B**) Analysis of MMP-3 and MMP-9 expression by qRT-PCR (****p < 0.0001, ***p < 0.001, **p < 0.01, *p < 0.05; ns = not significant).

The effect of the LINC00162 siRNA and sorafenib, both individually and in combination, on the migration of the B-CPAP cells was investigated by wound healing assay. While siRNA-mediated silencing of the LINC00162 lncRNA slightly reduced the migration ability of the B-CPAP cancer cells, sorafenib treatment led to a more significant reduction in migration compared to the control group. Furthermore, LINC00162 siRNA and sorafenib combination significantly decreased the migration of the B-CPAP cells, with a greater open wound area rate in this group compared to control (****p < 0.0001) and individually treated groups with LINC00162 siRNA (****p < 0.0001) and sorafenib (****p < 0.0001) (Fig. 10A).

To further confirm the findings from the wound healing assay, the expression levels of genes involved in cell migration and invasion were evaluated. The qRT-PCR results showed that the expression levels of MMP-3 (*p < 0.05) significantly decreased following LINC00162 silencing. Also, LINC00162 siRNA transfection reduced expression of the MMP-9 gene, however, this reduction was not significant compared to the control group. Sorafenib treatment significantly downregulated MMP-3 (**p < 0.01) and MMP-9 (*p < 0.05) expression compared to the control group. Following the combination of LINC00162 inhibition and sorafenib treatment, the relative mRNA expression of MMP-9 and MMP-3 decreased significantly compared to the control group and individual treatments. This confirms the findings from the wound healing assay (Fig. 10B).

### siRNA-mediated inhibition of LINC00162 LncRNAs combined with sorafenib inhibited MAPK pathway genes

**Fig. 11:**
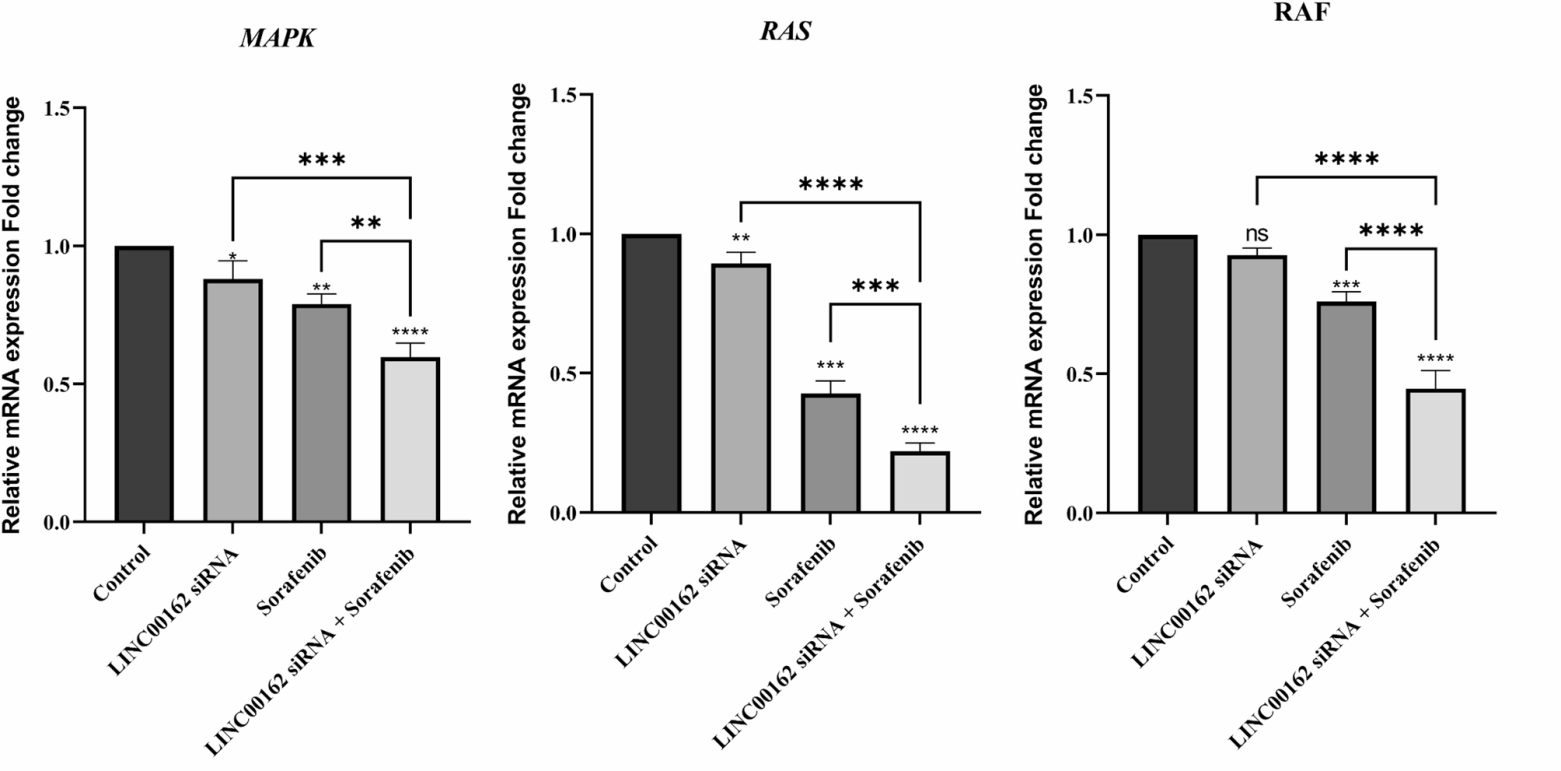
Analysis of the effect of LINC00162 siRNA transfection combined with sorafenib on the expression of MAPK, RAS, and RAF by qRT-PCR (****p < 0.0001, ***p < 0.001, **p < 0.01, *p < 0.05; ns = not significant).

Increased activation of the MAPK pathway is observed in thyroid cancer. Additionally, point mutations in the RAS and BRAF genes, which activate the MAPK pathway, were found in two-thirds of PTC cases. The kinase cascade pathway with RAS – RAF – MEK, and ERK is elevated following MAPK activation. In this context, we assessed the expression levels of the MAPK, RAF, and RAS gene expression. qRT-PCR results showed that siRNA-mediated silencing of the LINC00162 lncRNA significantly decreased expression of the MAPK (*p < 0.05) and RAS (**p < 0.01) genes, while the RAF levels did not change significantly. Sorafenib treatment also significantly decreased the expression of the MAPK, RAS, and RAF genes (**p < 0.01, ***p < 0.001, and ***p < 0.001, respectively) compared to the control group (Fig. 11). Furthermore, treating transfected cells with sorafenib further enhanced the suppression of MAPK, RAS, and RAF expression. These findings underscore the potential role of LINC00162 inhibition in downregulating the MAPK signaling pathway.

### siRNA-mediated Inhibition of LINC00162 LncRNAs combined with Sorafenib increased expression of the autophagic genes

**Fig. 12:**
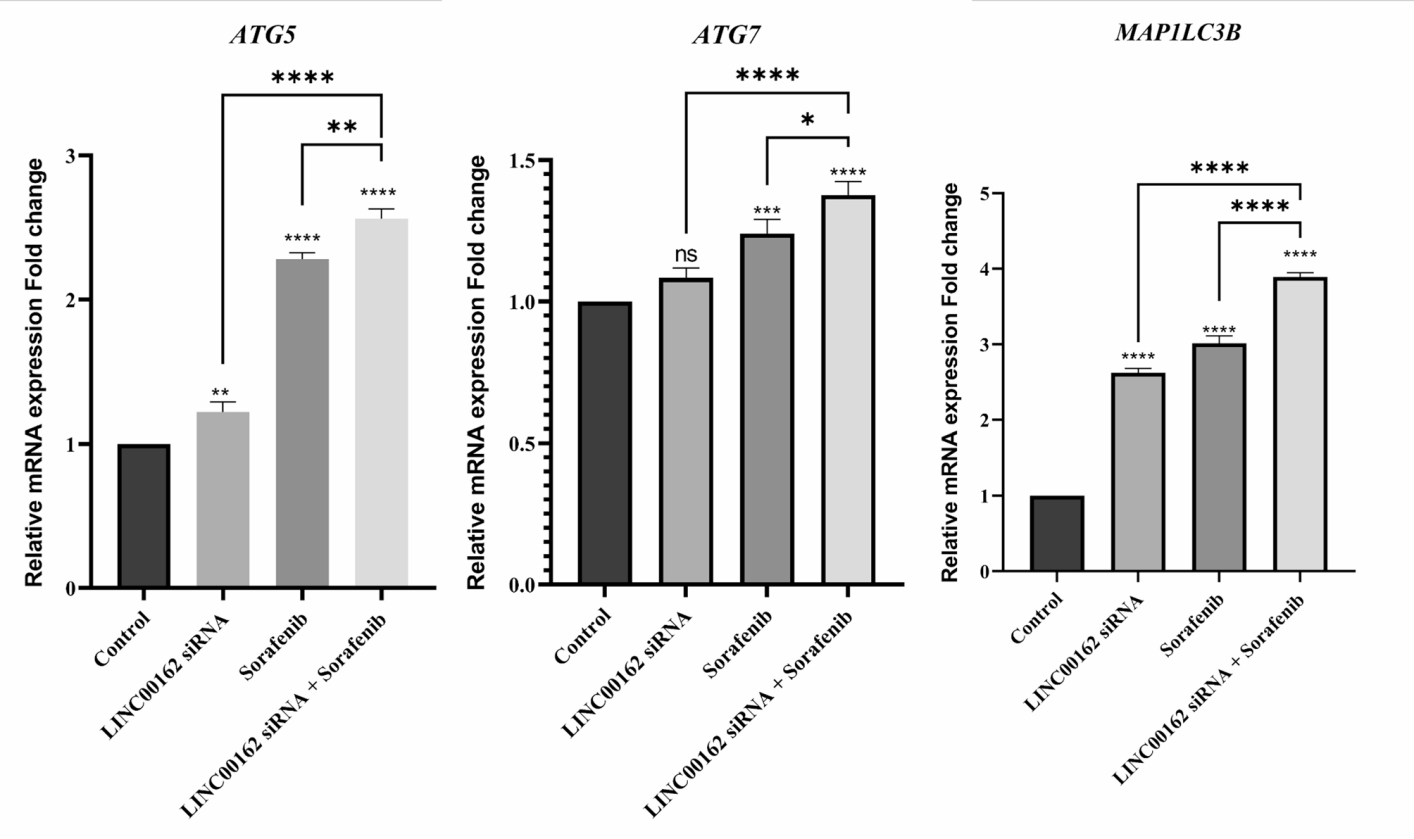
qRT-PCR analysis of the effect of siRNA-mediated silencing of the LINC00162 combined with sorafenib on the expression of ATG5, ATG7, and MAP1LC3B (****p < 0.0001, ***p < 0.001, **p < 0.01, *p < 0.05; ns = not significant).

To better understand the role of LINC00162 in thyroid cancer progression, we evaluated the expression of the autophagy-related genes ATG5, ATG7, and MAP1LC3B. Obtained results indicated that in the cells where LINC00162 was silenced by siRNA transfection, expression of ATG5 (**p < 0.01) and MAP1LC3B (****p < 0.0001) was significantly upregulated compared to the control group. However, no significant upregulation was observed in the expression of ATG7 in the LINC00162 siRNA-transfected group. Sorafenib treatment also significantly increased mRNA expression of the ATG5 (****p < 0.0001), ATG7 (***p < 0.001), and MAP1LC3B (****p < 0.0001). Furthermore, the qRT-PCR results showed that combining the LINC00162 siRNA and sorafenib treatment significantly upregulated ATG5, ATG7, and MAP1LC3B more than sole treatments (Fig. 12).

## Discussion

Thyroid cancer, which has a notable rise in its incidence in recent years, is the most common malignancy of the endocrine system^26^. Despite advancements in the diagnosis of thyroid cancer, approximately 6–20% of cases present regional or distant metastasis^27^. Papillary thyroid carcinoma (PTC) is the most prevalent histological type of thyroid cancer. Surgery, chemotherapy, radioactive iodine, and adjuvant radiation are the most common treatments for PTC^28,29^. Despite the generally favorable prognosis of PTC, some patients experience local recurrence or distant metastasis following surgery and radioactive iodine therapy^30^. PTC includes various tumor types that share mutations in genes responsible for encoding effectors that signal through the MAPK pathway. Among these, the BRAFV600E mutation has the highest prevalence^31^. However, the molecular mechanisms underlying PTC progression remain unclear, necessitating further research to identify novel pathways and therapeutic targets. In recent years, tyrosine-kinase inhibitors (TKIs), including sorafenib, a multikinase inhibitor, have been used in thyroid cancer treatment^32^. However, sorafenib does not significantly increase the survival rate due to side effects that lead to dose limitation^13^. Studies have shown that combining sorafenib with other molecular agents can enhance the treatment efficiency through synergistic effects^33^.

lncRNAs are emerging as key regulators in various stages of malignancy, and their oncogenic or tumor-suppressive functions have been demonstrated across different cancers^14^. Multiple studies have also reported the role of lncRNAs in thyroid cancer progression and metastasis. In a recent study, Shi et al. established that lncRNA GLTC promotes radioiodine resistance and progression of PTC by elevating LDHA activity through promoting the succinylation of LDHA at K155^34^. Li and coworkers also indicated that lncRNA SOCS2-AS1 increased PTC cell proliferation by promoting p53 degradation^35^. Considering the importance of lncRNAs, in this study, we investigated the effect of LINC00162 on the B-CPAP thyroid cancer cell line for the first time. Our earlier study demonstrated that LINC00162 is overexpressed in thyroid cancer tissue. In this study, we transfected LINC00162 siRNA and observed downregulation in the expression of LINC00162. We aimed to investigate the effect of the LINC00162 silencing on the chemosensitivity of B-CPAP thyroid cancer cells. MTT assay results indicated that LINC00162 silencing decreased the viability of the B-CPAP cells and increased the chemosensitivity of the B-CPAP cells to sorafenib by decreasing the IC50 from 11.48 µg/ml in sorafenib alone-treated cells to 7.760 µg/ml in cells transfected with LINC00162 and co-treated with sorafenib. Wang et al. investigated the effect of the PICSAR lncRNA on the sensitivity of the cutaneous squamous cell carcinoma cells to cisplatin. Their study revealed that PICSAR lncRNA sponges miR-485-5p in cisplatin-resistant cutaneous squamous cell carcinoma cells, which leads to REV3L overexpression and resistance of the cancerous cells to cisplatin. lncRNA PICSAR silencing reversed this pathway^36^. In another study conducted by Fei et al. overexpression of downregulated LncRNA-IQCH-AS1 sensitized thyroid cancer cells to doxorubicin. They reported that LncRNA contributed to the chemosensitivity of thyroid cancer cells by modulating the miR-196a-5p/PPP2R1B signaling pathway^21^. In addition to increasing the sensitivity of B-CPAP cancerous cells to sorafenib, flow cytometry analysis revealed that Linc00162 downregulation and sorafenib treatment induced apoptosis compared to the control group. Furthermore, a combination of Linc00162 siRNA and sorafenib remarkably evaluated the apoptosis rate. To reveal underlying molecular pathways, we assessed the expression of the BAX and BCL2 genes. Anti-apoptotic BCL2, which is an inner mitochondrial membrane protein, and its pro-apoptotic homolog, BAX, play an essential role in apoptosis^37^. Also, BCL2 expression confers protection to thyroid carcinomas by inhibiting apoptosis triggered by chemotherapy^38^. While siRNA-mediated silencing of LINC00162 and sorafenib treatment alone upregulated expression of BAX and downregulated Bcl-2 expression, qRT-PCR results showed a lower ratio of the BCL2/BAX in cells that received combination therapy compared to individual treatments and the control group. CASP3 and CASP9, which are cysteine proteases, play a key role in apoptosis induction in various cancers. Functioning as an initiator caspase, Caspase9 cleaves and activates Caspase3^39^. However, the role of caspase3 in thyroid cancer is not well defined. Therefore, we evaluated CASP3 and CASP9 expression to better understand the effect of the LINC00162 silencing on apoptosis induction in B-CPAP cells. Our results showed that expression of CASP3 and CASP9 noticeably increased following the siRNA-mediated downregulation of LINC00162. Also, combining the LINC00162 siRNA and sorafenib significantly upregulated these genes’ mRNA levels compared to individual treatments and control groups. Lee et al. demonstrated that silencing of LINC00162 induced apoptotic cell death. They reported that LINC00162 regulates apoptosis through sponging of miR-485-5p, and overexpression of miR-485-5p downregulates LINC00162. This miRNA suppresses PAQR4 expression. LINC00162 positively regulates PAQR4 which promotes proliferation and prevents apoptosis^40^. In terms of the role of other lncRNAs in PTC, Zhang et al. revealed the role of FOXD2-AS1/miR‐485‐5p/KLK7 in regulating apoptosis and cell proliferation in PTC cells. While FOXD2-AS1 lncRNA sponges miR-485-5p, upregulation of miR-485-5pincreases apoptosis rate. Also, knockdown of FOXD2‐AS1 and downregulation of KLK7 inhibited proliferation and induced apoptosis in PTC^41^. Furthermore, a study showed that silencing of FOXD2-AS1 lncRNA in B-CPAP cancer cells inhibited the survival of the cancer cells by increasing expression of Caspase3 and Caspase9^42^.

In addition to apoptosis, we also investigated the effect of LINC00162 silencing and sorafenib treatment on the cell cycle progression of B-CPAP thyroid cancer cells. Flow cytometry evaluation revealed that LINC00162 lncRNA silencing induced Sub-G1 cell cycle arrest, while sorafenib treatment induced arrest in both Sub-G1 and G2-M. The combination of LINC00162 silencing and sorafenib increased the percentage of arrested cells in the Sub-G1 and G2-M phases, suggesting the inhibitory role of LINC00162 silencing and combination therapy on B-CPAP cell progression. To investigate the mechanism responsible for the anti-proliferation effects of LINC00162 silencing, we evaluated the mRNA levels of MYC and TP53. qRT-PCR results showed that siRNA-mediated silencing of LINC00162 and sorafenib significantly downregulated MYC expression while upregulated TP53 expression in B-CPAP cells. Furthermore, in comparison to individual treatment groups, the combination therapy resulted in the lowest MYC expression levels and markedly enhancing the expression of TP53. c-Myc is an oncogenic transcription factor that promotes G1 to S-phase transition during the cell cycle^43^. One of the main ways that c-myc regulates the cell cycle is through inducing cyclin D2, which sequesters p27^44^. Cyclin D facilitates the initiation of the cell cycle by promoting the transition from the G0 or G1 phase to the S phase^45^. In this study, the molecular effects of LINC00162 on Cyclin D were also evaluated by qRT-PCR. Obtained results revealed that while LINC00162 silencing had no significant effect on expression of Cyclin D, its combination with sorafenib significantly increased inhibition of Cyclin D compared to the sole sorafenib treatment. These results confirm the anti-proliferative effect of LINC00162 silencing combined with sorafenib in B-CPAP PTC cells.

Furthermore, we evaluated the effect of LINC00162 silencing and its combination effect with sorafenib on B-CPAP cell invasion and colony formation ability. Results of the wound healing assay and colony formation assay indicated that while LINC00162 silencing slightly reduces migration and colony formation ability of cancer cells, its combination with sorafenib synergistically and significantly decreases migration, colony number, and the size of colonies. In alignment with our findings, in a study conducted on hepatocellular carcinoma cells, lncRNA PICSAR overexpression promoted colony formation while its knockdown inhibited the colony formation of the Hep3B cells^23^. Metastasis is a major cause of death in various cancers. Epithelial-mesenchymal transition (EMT) plays an important role in metastasis and contributes to drug resistance^46^. Matrix metalloproteinases (MMPs) contribute significantly to the EMT process and degrade ECM^47^. Considering the importance of MMPs in metastasis and invasion, we evaluated the expression of MMP-3 and MMP-9. Previous studies demonstrated increased expression of MMP-9 in thyroid cancer tissue and various thyroid cancer cell lines^48,49^. Our results demonstrated that the expression of MMP-3 decreased following siRNA-mediated silencing of LINC00162, independently of sorafenib. Also, treatment with sorafenib following LINC00162 silencing inhibited the expression of MMP-3 and MMP-9 more effectively compared to individual treatments and the control group.

Cancer stem cells (CSCs), which consist of a small population of cells, generate heterogeneous cells and retain the self-renewal ability of cancer cells. They are also resistant to chemotherapy drugs and regulate metastasis^50^. Increased expression of stemness markers, including SOX2, NANOG, CD133, and CD44, was reported in various thyroid cancer cell lines compared to normal thyroid^50^. To reveal the role of LINC00162 and sorafenib treatment in regulating the stemness of thyroid cancer cells, we evaluated the expression of SOX2, NANOG, CD133, and CD44 in B-CPAP cancer cells. qRT-PCR results showed that CD44, SOX2, and NANOG expressions were significantly downregulated in B-CPAP after LINC00162 silencing, while siRNA transfection did not have a significant impact on the expression of CD133. While sorafenib remarkably reduced the expression of these genes, the combined treatment was the most potent and inhibited the expression of CD44 and CD133 more effectively than the individual treatment. In a similar study, Li et al. demonstrated that in PTC, SOX2 induces LINC01510 transcription by binding to its promoter and increases its expression and knockdown of this lncRNA, suppressed the proliferation, migration, and invasion of thyroid cancer cells^51^. In summary, we proposed that LINC00162 silencing, either alone or combined with sorafenib, may impede thyroid cancer cell migration and metastasis by targeting the EMT process and related metastatic pathways.

MAPK signaling pathways play a crucial role in the development of various cancers^52,53^. It regulates proliferation, survival, metabolism, migration, invasion, and differentiation^53,54^. Alteration in MAPK signaling pathway components, including Ras, a molecular switch, is involved in PTC and activates MAPK^53,55^. A study published in 2025 reported overexpression of BRD9 in B-CPAP cells. They hypothesized that BRD9 promotes tumor growth in thyroid cancer by activating the MAPK/ERK signaling pathway. This gene may also promote the malignant phenotype of thyroid cancer cells and inhibit apoptosis by regulating c-Myc through activation of ERK^56^. Also, guanine-nucleotide exchange factors facilitate activation by Ras^57^. In our unpublished study, we demonstrated increased expression of the guanine nucleotide exchange factor, RAPGEFL1, in B-CPAP cells. siRNA-mediated silencing of the LINC00162 reduced the expression of the RAPGEFL1. To understand the underlying mechanisms involved in thyroid cancer progression, in this study, we evaluated the expression of the MAP, RAS, and RAF genes. The data displayed that lncRNA LINC00162 silencing significantly decreased MAPK and RAS expression, while it had no significant impact on RAF expression. Also, sorafenib treatment significantly reduced the expression of the MAPK, RAS, and RAF genes. Combination of LINC00162 silencing with multikinase inhibitor, sorafenib, remarkably increased inhibition of all three genes compared to individual treatments and the control group. Piipponen et al. demonstrated that p38 MAPK negatively regulates the expression of PICSAR in cutaneous squamous cell carcinoma^58^. These results indicate that the knockdown of LINC00162 decreased RAPGEFL1 expression, may leading to RAS activation and downregulation of the MAPK pathway. Current findings suggest that LINC00162 may influence the MAPK pathway, and sorafenib promotes this regulatory effect. Further research needs to be conducted.

Autophagy, as a cellular mechanism that destroys organelles and proteins, can promote or inhibit cancer progression and regulate metastasis^59–61^. ATG family genes, including ATG5, and LC3B (ATG8F) regulate autophagy by being involved in autophagosome formation^62^. ATG7, an E1 enzyme, plays a key role in initiating autophagy by guiding ATG12 and ATG8 to their respective E2 enzymes^63^. To investigate the impact of LINC00162 knockdown and sorafenib treatment on autophagy in thyroid cancer cells, we analyzed the expression levels of ATG5, ATG7, and MAP1LC3B genes in B-CPAP cancer cells. qRT-PCR results showed that ATG5 and LC3B expressions were significantly upregulated in B-CPAP after LINC00162 silencing, while siRNA transfection did not have a significant impact on the expression of ATG7. Although sorafenib significantly elevated the expression of these genes, the combined treatment proved to be the most effective, further enhancing the expression of ATG5, ATG7, and MAP1LC3B compared to sole treatments and control groups. Another study demonstrated that RBM47 upregulation, which is downregulated in PTC, regulates FOXO3, thereby promoting the transcription of ATG3 and ATG5 and activation of autophagy in PTC. They also observed a reduction in PTC progression following RBM47 upregulation^64^. Qin et al. demonstrated that GAS8-AS1 LncRNA, which is downregulated in PTC cells, has a tumor-suppressive role and activates autophagy by increasing the expression of ATG5 and leading to cell death^65^.

While this study’s findings are promising, some limitations need to be addressed. One of the main limitations associated with systemic application of siRNAs in humans is the presence of RNase A in serum, which can degrade siRNA before it reaches its cellular destination^66^. Another unsolved issue is the specific and efficient delivery of the siRNA for therapeutic purposes. Systemically administered siRNAs primarily accumulate in the liver. Delivering sufficient siRNA and achieving therapeutic concentrations of siRNAs in other tissues remain a significant challenge. For cellular uptake, siRNAs must be packaged into stable nanoparticles because they cannot readily diffuse across cell membranes. Early studies reported siRNA as specific and non-immunogenic. However, further studies reported off-target activity of siRNAs. This off-target activity can lead to unanticipated phenotypes, hinder the accurate assessment of the therapeutic efficacy of siRNAs, and lead to unwanted toxicities^67–69^. Conducting more studies in the future may help overcome these limitations. Additionally, investigating the effect of LINC00162 combined with sorafenib in animal models, assessing protein levels, and conducting complementary in vitro tests may enhance treatment for PTC patients.

## Conclusion

We investigated the effect of the siRNA-mediated downregulation of LINC00162 and its combination with sorafenib on B-CPAP thyroid cancer cells for the first time. Taken together, our results revealed that LINC00162 has an oncogene role, and its knockdown increased the sensitivity of the B-CPAP cells to sorafenib. Also, apoptosis and sub-G1 cell cycle arrest were induced following LINC00162 silencing. In addition to inhibiting the migration and ability to form colonies, LINC00162 silencing also downregulated the expression of the genes involved in migration and stemness. Furthermore, our study revealed the role of LINC00162 silencing in inhibiting thyroid cancer progression through decreasing the expression of the MAPK, RAS, and RAF genes. Furthermore, LINC00162 silencing, combined with sorafenib, significantly reduced the viability, progression, and stemness ability of the B-CPAP cells. In conclusion, silencing LINC00162 and also its combination with sorafenib could play a role in thyroid cancer treatment and improve patient survival.

## Data Availability

all data generated or analysed during this study are included in this submitted manuscript.
